# An interpretable and interactive deep learning algorithm for a clinically applicable retinal fundus diagnosis system by modelling finding-disease relationship

**DOI:** 10.1038/s41598-023-32518-3

**Published:** 2023-04-12

**Authors:** Jaemin Son, Joo Young Shin, Seo Taek Kong, Jeonghyuk Park, Gitaek Kwon, Hoon Dong Kim, Kyu Hyung Park, Kyu-Hwan Jung, Sang Jun Park

**Affiliations:** 1 VUNO Inc., Seoul, Republic of Korea; 2 Department of Ophthalmology, Seoul Metropolitan Government Seoul National University Boramae Medical Center, Seoul, Republic of Korea; 3 Department of Ophthalmology, College of Medicine, Soonchunhyang University, Cheonan, Republic of Korea; 4 Department of Ophthalmology, Seoul National University College of Medicine, Seoul National University Bundang Hospital, 82, Gumi-ro 173 Beon-gil, Bundang-gu, Seongnam-si, Gyeonggi-do 13620 Republic of Korea; 5 Department of Medical Device Research and Management, Samsung Advanced Institute for Health Sciences and Technology, Sungkyunkwan University, 81 Irwon-ro, Gangnam-gu, Seoul, Republic of Korea

**Keywords:** Biomedical engineering, Eye abnormalities, Computational models, Machine learning

## Abstract

The identification of abnormal findings manifested in retinal fundus images and diagnosis of ophthalmic diseases are essential to the management of potentially vision-threatening eye conditions. Recently, deep learning-based computer-aided diagnosis systems (CADs) have demonstrated their potential to reduce reading time and discrepancy amongst readers. However, the obscure reasoning of deep neural networks (DNNs) has been the leading cause to reluctance in its clinical use as CAD systems. Here, we present a novel architectural and algorithmic design of DNNs to comprehensively identify 15 abnormal retinal findings and diagnose 8 major ophthalmic diseases from macula-centered fundus images with the accuracy comparable to experts. We then define a notion of counterfactual attribution ratio (CAR) which luminates the system’s diagnostic reasoning, representing how each abnormal finding contributed to its diagnostic prediction. By using CAR, we show that both quantitative and qualitative interpretation and interactive adjustment of the CAD result can be achieved. A comparison of the model’s CAR with experts’ finding-disease diagnosis correlation confirms that the proposed model identifies the relationship between findings and diseases similarly as ophthalmologists do.

## Introduction

Ophthalmologists and primary care practitioners often examine macula-centered retinal fundus images for comprehensive screening and efficient management of vision-threatening eye diseases such as diabetic retinopathy (DR)^1^, glaucoma^2^, age-related macular edema (AMD)^3^, and retinal vein occlusion (RVO)^4^. Deep learning (DL) algorithms^5^ have been developed to automate the assessment of DR^6,7^, glaucoma^8,9^, and AMD^10,11^, as well as multiple ophthalmologic findings^12^, achieving performance comparable to that of human experts. A major obstacle hindering the applicability of DL-based computer-aided diagnosis (CAD) systems in clinical setting is its interpretability, that is, the rationale behind its diagnostic conclusions is obscure. Several visualization techniques such as class activation maps^13,14^ and integrated gradients^15^ have been developed to highlight lesions as preliminary solutions. However, the ‘heatmap’ provides only ambiguous regions on the image that contributed to the final prediction and cannot explicitly differentiate the lesions that attributed to the final model prediction. Therefore, the users may not fully understand which findings contributed to the DL system’s diagnostic predictions. Another limitation of preexisting DL-based algorithms for fundus image analysis is that they are capable of examining only a few ophthalmologic findings or diseases (e.g., DR), while more comprehensive coverage of multiple common abnormal retinal conditions is necessary for practical deployment of DL-based CAD systems to clinical settings.

We present a DL-based CAD system that not only comprehensively identifies multiple abnormal retinal findings in color fundus images and diagnoses major eye diseases, but also quantifies the attribution of each finding to the final diagnosis. The training procedure resembles ophthalmologists’ typical workflow, first identifying abnormal findings and diagnosing diseases based on the findings present in the fundus image. This DL system presents the final diagnostic prediction and their accompanying heatmap just as other available DL systems, and also provides the quantitative and explicit attributions of each finding in making the proposed diagnoses, which enhances the interpretability of the provided diagnostic decision, to the benefit of physicians in making their final decisions for the right treatment or management of ophthalmic diseases. The model’s performance was validated on a held-out, in-house dataset as well as 9 external datasets. A novel notion of counterfactual attribution ratio (CAR) was used to elucidate the rationale behind our DL system’s decision-making process by quantifying the extent to which each finding contributes to its diagnostic prediction. Statistical analysis of CAR was performed to evaluate if the DL system’s clinical relations between finding identification and disease diagnoses were similar to that of human experts.

## Results

### Reliability of the DL-system

**Figure 1:**
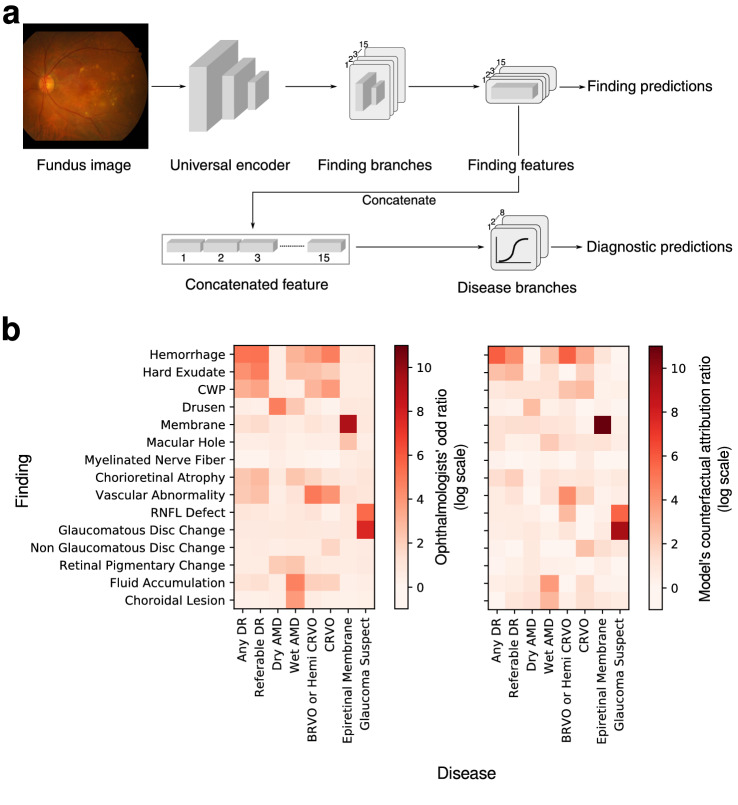
(**a**) Overall architecture of the deep learning-based model. (**b**) Relationships between ophthalmolgic findings and eye diseases computed by aggregate annotations of human experts and the model’s counterfactual attribution ratio.

The system consists of two major components that are implemented in a single neural network: (1) *fifteen-finding identification subnetwork* is specialized to predict the likelihood that each finding is present in a fundus image, and (2) *eight-disease diagnosis subnetwork* diagnoses retinal diseases based on features extracted from the finding-identification network (Fig. 1a). The 15 findings considered in this system consist of hemorrhage, hard exudate, cotton wool patch (CWP), drusen, membrane, macular hole, myelinated nerve fiber, chorioretinal atrophy or scar, any vascular abnormality, retinal nerve fiber layer (RNFL) defect, glaucomatous disc change, non-glaucomatous disc change, fluid accumulation, retinal pigmentary change, and choroidal lesion. The 8 major diseases considered are dry AMD, wet AMD, any DR, referable DR, central retinal vein occlusion (CRVO), branch retinal vein occlusion (BRVO)/hemi-CRVO, epiretinal membrane, and glaucoma suspect.^16^.

The finding-identification models and disease diagnosis models were trained and tested on data of 103,262 macula-centered fundus images from 47,764 patients (Supplementary Table 1). All models were evaluated with respect to its area under the receiver operating characteristic curve (AUROC), with its 95% confidence interval computed using the Clopper-Pearson method. Operating points were chosen to maximize the harmonic mean between sensitivity and specificity (i.e. F1-score) on the in-house validation data set except for BRVO/hemi-CRVO and CRVO whose operating points were set to have approximately 90% sensitivity because only a small number of positive cases were available.

### Component #1: identification of fifteen abnormal findings

As shown in Table 1, the system identified findings in retinal fundus images with a mean AUROC of 0.980 across all 15 findings on a held-out, in-house test set. AUROCs ranged from a minimum of 0.972 for retinal pigmentary change to a maximum of 1.000 for myelinated nerve fiber layer with respect to the majority consensus of three ophthalmologists as reference. Sensitivities ranged from 92.5% for glaucomatous disc change to 100.0% for myelinated nerve fiber and specificity varied between 88.3% for retinal pigmentary change to 100.0% for myelinated nerve fiber (Supplementary Table 2, Supplementary Fig. 1). This performance is comparable to human experts as reported in previous literature.^12^.

**Table 1:** Area under receiver operating characteristic curves of the proposed models on the in-house test dataset and external datasets.

	In-house test set	External dataset	External datasets[Source]
	SNUBH test set	MESSIDOR	
Normality			
Abnormal fundus	0.980 (0.969–0.988)	0.915 (0.876–0.943)	–
Finding			
Hemorrhage	0.995 (0.982–0.999)	0.950 (0.913–0.973)	0.979 (0.855–0.999) [e-ophtha], 0.985 (0.805–1.000) [IDRiD]
Hard exudate	0.990 (0.972–0.998)	0.971 (0.929–0.992)	0.964 (0.661–0.998) [e-ophtha], 1.000 (0.692–1.000) [IDRiD]
Cotton wool patch	0.998 (0.979–1.000)	0.900 (0.848–0.940)	0.991 (0.735–1.000) [IDRiD]
Drusen	0.985 (0.973–0.994)	0.866 (0.810–0.908)	–
Membrane	0.997 (0.985–1.000)	0.954 (0.881–0.991)	–
Macular hole	0.993 (0.974–0.999)	1.000 (0.872–1.000)	–
Myelinated nerve fiber	1.000 (0.985–1.000)	1.000 (0.884–1.000)	0.999 (0.541–1.000) [STARE]
Chorioretinal atrophy	0.997 (0.985–1.000)	0.972 (0.905–0.997)	–
Vascular abnormality	0.993 (0.978–0.999)	0.936 (0.849–0.989)	–
RNFL defect	0.986 (0.970–0.996)	0.895 (0.797–0.957)	–
Glaucomatous disc change	0.981 (0.963–0.991)	0.985 (0.874–0.999)	–
Non-glaucomatous disc change	0.977 (0.956–0.989)	0.865 (0.721–0.947)	–
Retinal pigmentray change	0.972 (0.954–0.985)	0.804 (0.711–0.872)	–
Fluid accumulation	0.980 (0.958–0.993)	0.869 (0.737–0.951)	–
chorodial lesion	0.989 (0.967–0.998)	0.892 (0.782–0.967)	–
Diagnosis			
Any DR	0.997 (0.984–1.000)	0.957 (0.919–0.982)	0.999 (0.988–1.000) [APTOS], 0.947 (0.879–0.979) [IDRiD]
Referable DR	0.999 (0.987–1.000)	0.983 (0.946–0.998)	0.974 (0.958–0.985) [APTOS], 0.973 (0.907–0.997) [IDRiD]
Dry AMD	0.984 (0.969–0.992)	0.938 (0.863–0.976)	0.936 (0.845–0.977) [ADAM]
Wet AMD	0.997 (0.981–1.000)	–	
BRVO/Hemi-CRVO	0.992 (0.975–0.999)	0. 961 (0.842–0.999)	0.965 (0.828–0.999) [STARE]
CRVO	0.994 (0.970–0.999)	–	0.935 (0.792–0.992) [STARE]
Epiretinal membrane	0.997 (0.984–1.000)	0.970 (0.889–0.999)	0.984 (0.478–1.000) [STARE]
Glaucoma suspect	0.979 (0.963–0.990)	0.945 (0.851–0.989)	0.951 (0.792–0.992) [REFUGE-train], 0.967 (0.880–0.999) [REFUGE-val,test]

If no positive cases exist, the cells are filled with “–”.

*AMD* Age-related macular degeneration, *BRVO* Branch retinal vein occlusion, *CRVO* Central retinal vein occlusion, *DR* Diabetic retinopathy, *RNFL* Retinal nerve fiber layer.

The models were then validated on 4 external datasets without additional tuning to identify findings included in each dataset: MESSIDOR^17^ $\left( {n = 1189} \right)$ for all 15 findings, e-ophtha^18^ $\left( {n = 434} \right)$ for hemorrhage and hard exudate, IDRiD-segmentation^19^ $\left( {n = 143} \right)$ for hemorrhage, hard exudate, and CWP, as well as STARE^20^ $\left( {n = 397} \right)$ for the presence of myelinated nerve fiber. On the MESSIDOR dataset, which consists of external images internally annotated by three retinal specialists (PSJ, SJY, KHD), the model achieved an average AUROC of 0.915 with a minimum of 0.804 for retinal pigmentary change to a maximum of 1.0 for macular hole and myelinated nerve fiber layer. Sensitivity on the MESSIDOR dataset was rather compromised ranging from 42.5% for drusen to 100.0% for myelinated nerve fiber and macular hole while specificity was comparable to that of the in-house test set ranging from 88.3% for hard exudate to 100.0% for myelinated nerve fiber. On external datasets from multiple sources, the model attained AUROCs ranging from 0.964 (e-ophtha, hard exudate) to 1.000 (IDRiD, hard exudate). Sensitivity was comparable to that of the in-house test set with a minimum of 85.1% (e-ophtha, hemorrhage), but specificity dropped in certain datasets down to a minimum of 65.7% (e-ophtha, hard exudate).

### Component #2: diagnosis of eight major eye diseases

**Figure 2:**
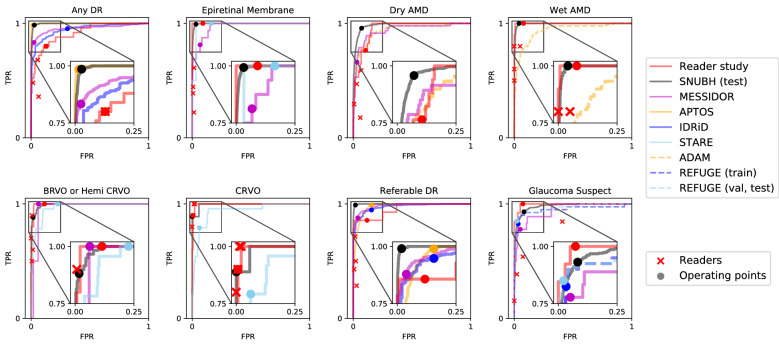
Receiver operating characteristic curves for diseases. Gray boxes are amplified to present the profile of the curves in a practical range. Operating points of the models are marked with circles and performance of human readers are plotted with cross marks. Receiver operating characteristic curves for ADAM dataset is identical for dry AMD and wet AMD.

The diagnostic performance of the disease models trained on top of the finding models reached a mean AUROC of 0.992 across all 8 diseases in the in-house, held-out test set, ranging from 0.979 for glaucoma suspect to 0.999 for referable DR (Table 1 and Fig. 2). This performance carried on to the MESSIDOR and all 6 external datasets: MESSIDOR ($n = 1189$) with an average AUROC of 0.959 ranging from 0.938 for dry AMD to 0.983 for referable DR, Kaggle APTOS-2019 challenge $\left( {n = 3662} \right)$ with 0.986 AUROC averaged over DR and referable DR, IDRiD-classification^19^ $\left( {n = 3662} \right)$ with average AUROC of 0.968 for the same diseases, 0.951 and 0.967 for glaucoma suspect on REFUGE^21^ (training) $\left( {n = 400} \right)$ and REFUGE^21^ (val, test) $\left( {n = 800} \right)$ respectively, 0.943 for dry and wet AMD on ADAM^22^ $\left( {n = 400} \right)$, and 0.964 averaged across CRVO, BRVO/hemi-CRVO, and epiretinal membrane on STARE $\left( {n = 397} \right)$. The disease diagnosis models’ receiver operating characteristic curves in Fig. 2 are comparable to that of four physicians’ operating points, demonstrating their practicality as a comprehensive CAD system. However, as observed for the finding models, diagnostic models exhibited either exclusively high sensitivity or specificity on some datasets with a minimum sensitivity of 77.8% (MESSIDOR, glaucoma suspect) and minimum specificity of 69.0% (IDRiD, DR).

### Quantification of clinical relations between findings and diseases

**Figure 3:**
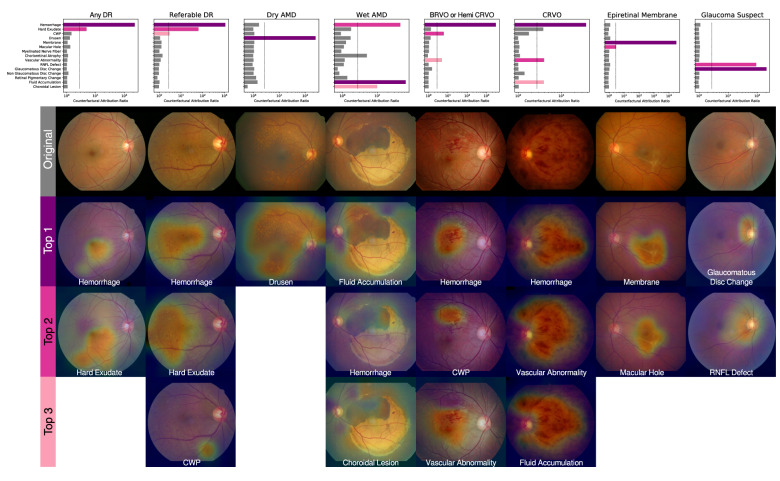
Use cases of counterfactual attribution ratio in diagnostic fundus examination. Lesions of top 3 findings above a threshold (natural constant *e*) are shown (attribution activation map).

The unique finding-disease network architecture permits the use of a newly defined notion of CAR of each finding-disease pair as detailed in the “Methods” Section. CAR quantifies the extent to which the model’s diagnostic prediction attributes to a finding by comparing the disease diagnosis model’s predictions under two hypothetical situations in which the finding of interest had been present or absent with absolute confidence. This notion is directly comparable to the odds ratio calculated for human experts, and can be interpreted as a measure of the clinical relation between finding-disease pairs. The similarity of CARs for all finding-disease pairs from our CAD system and odds ratio collectively estimated over 57 board-certified ophthalmologists on the in-house test dataset demonstrates how both entities generate similar diagnostic opinions (Fig. 1b). The network associated diseases with findings as following: DR and referable DR with hemorrhage and hard exudate that are key clinical findings used to diagnose the severity of DR; dry AMD with drusen; wet AMD with fluid accumulation, choroidal lesion, and hemorrhage; RVO with hemorrhage, CWP, vascular abnormality; epiretinal membrane with membrane but not macular hole, although epiretinal membrane and macular hole frequently occur together as shown in the human’s odds ratio; glaucoma suspect with glaucomatous disc change and RNFL defect. The agreement between the model’s CAR values and human experts’ odds ratio reveals the similar attribution of identified findings in diagnostic predictions as in medical practice. Figure 3 illustrates how our CAD system can visualize the model’s attribution ratio between finding-disease pairs.

**Figure 4:**
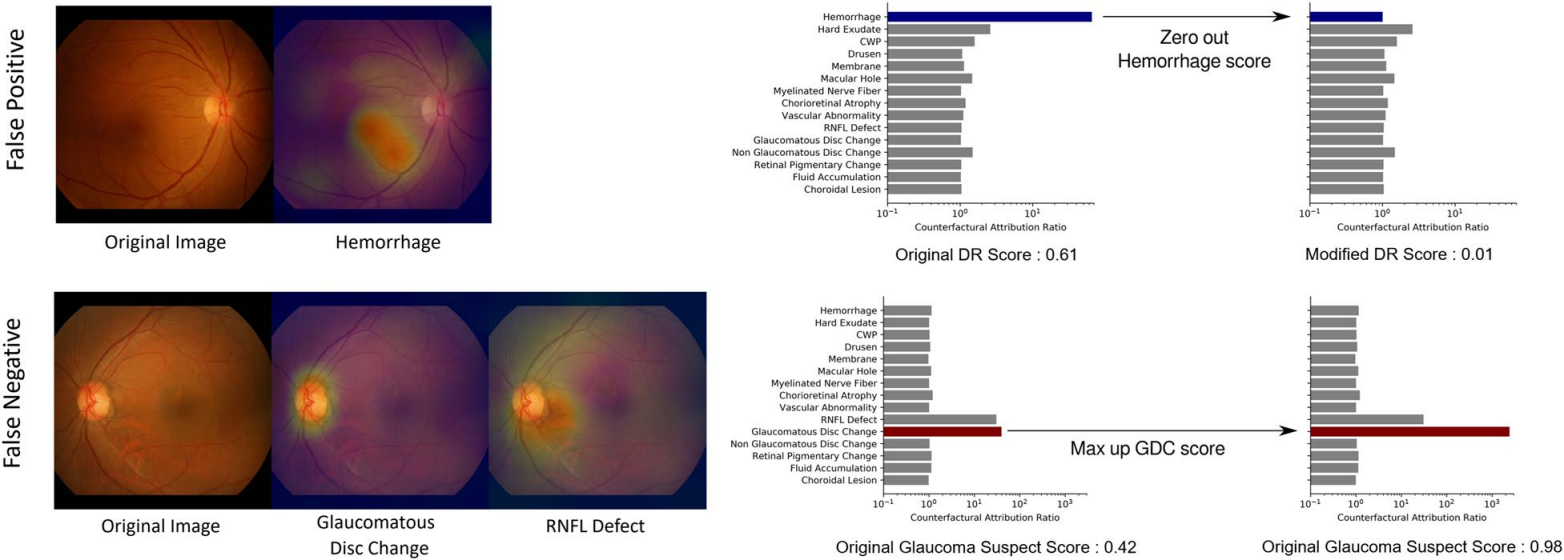
Interactive diagnosis using counterfactual attribution ratio and the attribution maps of relevant findings. (top) A false-positive diagnosis of diabetic retinopathy can be corrected by suppressing the overestimated attribution of hemorrhage. (bottom) A false-negative diagnosis of glaucoma can be corrected by increasing the underestimated attribution of glaucomatous disc change.

To compensate for the inevitable imperfection of deep neural networks (DNNs), a CAD system should ideally be interactive when readers disagree with the model’s finding identification or diagnostic prediction partially or entirely. Figure 4 shows how a reader can interact with this CAD system to reduce or increase the influence of a certain finding on the model’s diagnostic prediction, by modifying the finding’s prediction score between values of 0 and 1. For example, if the reader thinks the model missed the diagnosis of glaucoma suspect due to underestimation of the presence of glaucomatous disc change, the reader may simply modify the attribution of glaucomatous disc change to 1 and obtain its corresponding diagnostic prediction of glaucoma suspect. This ‘physician-in-the-loop’ procedure does not require an additional inference step and the re-evaluation incurs negligible computation. This is especially useful when the mis-identification is due to sensor error such as a stain on the camera’s lens identified as hemorrhage or CWP^15^. The interactive diagnosis enabled by the use of the CAR framework ultimately makes the models’ diagnostic predictions readily adaptable to noise or ambiguity pervasive in practical clinical settings.

### Hemorrhage feature maps for various different diagnoses

**Figure 5:**
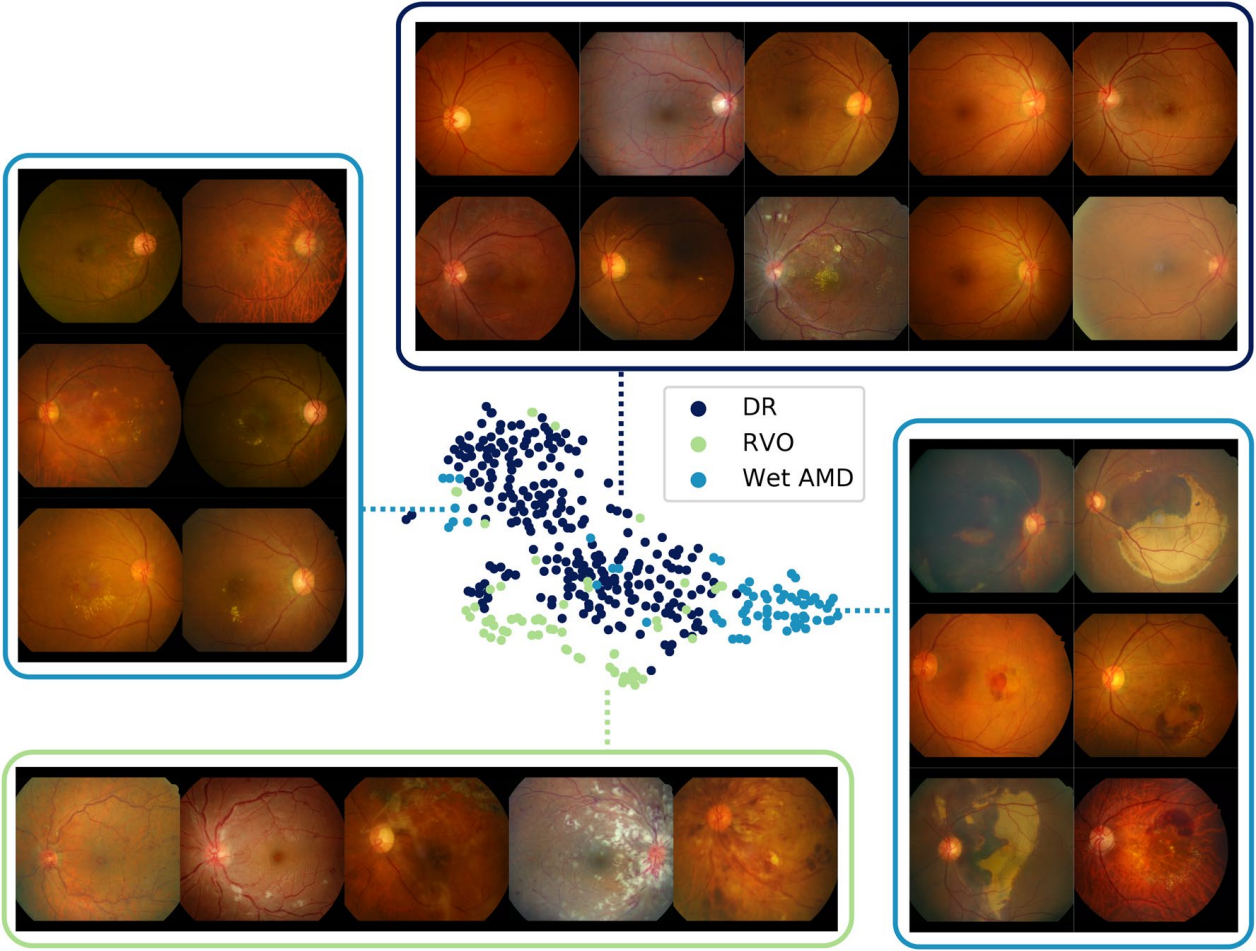
t-Distributed Stochastic Neighbor Embedding plots of hemorrhage feature maps for positive cases of diseases that involve hemorrhage in diagnostic decisions.

During the annotation process, various types of one finding were grouped into a broader super-class to enhance simplicity in the model and also for efficiency in obtaining large-scale labeled datasets^12,16^. For example, various sub-types of hemorrhage such as flame-shaped hemorrhage, dot hemorrhage, splinter hemorrhage, blot hemorrhage, subretinal hemorrhage, preretinal hemorrhage, vitreous hemorrhage were all classified as one super-class hemorrhage; however, a fine-grained classification of the findings critically effects diagnostic conclusions. A model trained to classify the super-class may be unable to discriminate precise patterns corresponding to different sub-findings and consequently yield incorrect diagnostic predictions. Figure 5 visualizes the latent spaces of different sub-features by projecting features extracted from the in-house test set using t-Distributed Stochastic Neighbor Embedding (t-SNE). Feature maps for hemorrhage were extracted for positive cases of DR, RVO, and wet AMD, where positive cases were defined as an agreement of more than two independent annotators. Three clusters in large represent each disease showing different patterns of hemorrhage. Samples of DR show dot hemorrhage and blot hemorrhage in localized areas, and RVO cases include flame-shaped hemorrhage in a broad area while intraretinal and subretinal hemorrhage of all shapes and sizes are possible in cases of wet AMD. Several outliers for wet AMD on the left side of the t-SNE plot comprise small amounts of bleeding, resembling blot hemorrhage. From this, it is evident that although the model does not have access to fine-grained specifications of types of sub-findings, different sub-categories of hemorrhage and their corresponding patterns are well-preserved.

## Discussion

A DL-based retinal fundus screening algorithm that comprehensively identifies abnormal retinal findings and diagnose major eye diseases performed comparably to expert ophthalmologists on several external datasets. The proposed CAR scheme empowers the user to interpret the model’s predictions, as clinical relations between its diagnostic predictions and identified findings can be quantified and visualized. A comparison of the model’s CAR values with ophthalmologists’ odds ratios measuring how experts associate diseases with individual findings demonstrates the similarity in how the model draws diagnostic conclusions from associated abnormal findings. Owing to the model’s transparency, physicians can reliably use our DL-based CAD system to make diagnostic predictions assured by the fact that predictions are primarily based on findings identified in the fundus image. Moreover, a user who disagrees with the model’s prediction of whether a finding is present or absent can freely adjust the finding prediction and re-evaluate the diagnostic prediction with negligible computational cost.

Prior works have reported outstanding standalone performance of DL-based models in classifying presence of individual ocular diseases in macula centered fundus images. Gulshan et al.^6^ achieved an AUROC of 0.99 in discriminating referable DR on validation datasets which was comparable to ophthalmologists. Ting et al.^7^ developed DL systems for DR, AMD and glaucoma, and showed comparable performance to human experts achieving high AUROCs ranging from 0.94 to 0.96. Li et al.^8^ also reported a high AUROC of 0.99 for glaucomatous optic neuropathy on their validation dataset. Burling et al.^10^ achieved AUROCs of 0.94 to 0.96 for AMD classification scaling the severity of AMD on in-house cross-validation datasets and DeepSeeNet^11^, and achieved an AUROC of 0.97 for detecting late AMD.

In attempt to understand the reasoning behind convolutional neural networks, Taylor decomposition^23^ and Shapley value estimation^24^ have been applied to the development of various diagnostic CAD systems^25^. Nevertheless, their objective is to acquire a precise heatmap illustrating the feature’s attributions. On the other hand, our system identifies explicit and interpretable reasoning behind disease predictions using finding predictions from training from a large-scale annotated dataset. Another line of work instead retrieves images to attribute the model’s predictions to a similar image from a fixed pool, measuring their proximity as the Euclidean distance between the test and candidate images’ features^26^. This has been used to interpret the reasoning behind an algorithm diagnosing acute intracranial hemorrhage by displaying similar patterns of images used for training^27^.

Kim et.al proposed to utilize a normal vector of a decision boundary for a certain type of pattern, or a concept, to measure the attribution of patterns to the final prediction^28^. Technically, they measured the impact of changes along the direction of the normal vector to a score of a certain class, where normal vectors are derived at multiple hidden layers. CAR parallels with the work of Kim et al. when findings are concepts and classes are modified into multiple binary decision for diseases. However, CAR complements this work in two key aspects. First, CAR provides exact quantitative values for the attribution in the form of odds ratio, and a direct comparison with domain-specific knowledge is possible. In our case, the exact results of CAR allowed us to compare it to a collective odds ratio of human experts in a large-scale dataset. Also, CAR relies on normal vectors of the decision boundaries at one embedding or feature space hence reducing computation. Our experimental results suggest that concepts at the embedding of the last layer of convolutional neural network suffice for attribution analyses.

Also, a linear projection of finding features to disease diagnostic predictions did not compromise performance, when comparing its validation and test performances with end-to-end (Image to Disease) architectures (Supplementary Table 3). Our disease network’s architecture performing on par with deep, end-to-end architectures shows how finding features convey the overall pathological irregularities and even the simplest regression diagnostic model can reach holistic conclusions based on finding features. Predicting various diseases with shallow additional layers on top of finding features significantly reduces memory and computation, and our finding-disease architecture is expected to be more widely applicable especially in resource-tight environments including rural clinics or heavily-loaded cloud systems. Moreover, the simple architecture can be fine-tuned more easily with newly available data as only the last layer’s parameters need to be modified.

CAR only requires that the training of classification models on findings and diseases are separated into two phases, and is thus applicable to other medical modalities including chest radiographs. To this end, we assessed CAR on chest X-ray (CXR) modalities by training finding identification models to identify the following radiographic findings: nodule/mass, consolidation, interstitial opacity, pleural effusion, and pneumothorax. Disease diagnostic models were trained on the feature extracted using finding identification models as done previously to classify tuberculosis (TB), edema, and pneumonia. All models performed reliably, and applying CAR revealed that the model agrees with clinical knowledge, associating TB with nodular and consolidation patterns, edema with interstitial opacity and pleural effusion, and pneumonia with consolidation patterns (Supplementary Fig. 2).

It is widely known that DNNs suffer from an opaque low-dimensional representation of high dimensional data. Typical qualitative analysis of its resulting latent space includes visualization using techniques such as t-SNE. Alternatively, CAR admits a quantification of clinical relations between findings and diseases, and their agreement with known clinical association enables a quantitative and interactive analysis of whether the model learnt a clinically-meaningful representation of medical data. If not, it is possible to identify diagnostic bias. From our experiments on diagnosing DR as in Fig. 1b, we were able to infer that the model was not presented with enough CWP or vascular abnormality (e.g., neovascularization) data throughout training. Conclusively, CAR testifies the limitations of the underlying classification models.

In the light of interest aimed at building comprehensive neural networks that identify abnormal findings of retinal fundus images and their corresponding diagnoses, our algorithmic design and proposed CAR ameliorates two properties of existing DL that repel their clinical usage as CAD systems: interpretability and reliability. In addition to evaluating a model’s performance on a test set, one can verify whether the model behaves similarly to clinical practice by comparing their CAR scores with the odds ratio of domain experts that measure how findings atribute to diagnostic decisions in clinical practice. The illuminative interpretability and enhanced reliability of DNNs provided by this CAD system can be paramount for real-world deployment.

## Methods

### Developmental environment

All experiments were conducted on a server with 4 TITAN X (Pascal) GPUs, 48 Intel (R) Xeon (R) CPUs (E5-2670 v3@2.30 GHz) and 256 GB memory using the DL frameworks Tensorflow 1.14.0 and Keras 2.2.5.

### In-house dataset

The retinal fundus images used to train and test the finding-identification networks were acquired from the health screening center and ophthalmology outpatient clinic at the Seoul National University Bundang Hospital (SNUBH) from June 1st, 2003 to June 30th, 2016 using various fundus cameras (CF60Uvi, CR6-45NM [Canon, Utsunomiya, Japan], VX-10, VX-10α, nonmyd 7, GENESIS-D [Kowa Optimed, Tokyo, Japan], etc.)^16^ Fifty seven board certified ophthalmologists (of which 28 were certified specialists; 16 certified retina specialists, 9 certified glaucoma specialists, and 3 certified cornea specialists) annotated the fundus images in accordance to the guidelines (Supplementary Table 4). The study was conducted in accordance with the tenets of the Declaration of Helsinki, and the data used was approved by the institutional review board at Seoul National University Bundang Hospital (IRB No. B-1508-312-107). Written consent was waived by the institutional review board due to the retrospective nature of the study. All subjects were de-identified by randomly assigning 10-digits and removing all personal data (e.g. patient name, birthdate, study date) except gender and age at the time of the study. All data specifications are summarized in Supplementary Table 1.

Subjects were randomly divided into three groups without overlaps: train (85%), validation for hyperparameter tuning (5%), and test (10%) sets, and fundus images corresponding to each subject were assigned to the corresponding groups.

### Annotation of retinal fundus images

A pool of fifty seven ophthalmologists assessed fundus images concerning image quality, abnormality, findings, and diagnoses in a stepwise manner, using an annotation tool dedicated to large-scale data collection^12,16^. Each fundus image was randomly assigned to three readers for assessment. Fundus images assessed abnormal were additionally annotated at the lesion-level for all fifteen abnormal retinal findings, and readers reported comprehensive diagnostic opinions regarding eight eye diseases^16^. During the assessment, readers were informed of gender and age. Each image was assessed independently by three different readers, yielding a total of 309,786 readings for 103,262 fundus images. Findings and diseases were categorized in a way that reflects prevalent ophthalmic abnormalities in primary care clinics and health screening centers. Specifically, the subcategories corresponding to each finding (e.g., flame hemorrhage, disc hemorrhage, microaneurysm) were grouped into a single category (e.g., hemorrhage), and subtypes of diseases were categorized in accordance with internationally accepted guidelines as follows: as for AMD, dry AMD includes early AMD and geographic atrophy while wet AMD includes cases with choroidal neovascularization. For DR, non-referable DR was mild non-proliferative DR while referable DR included moderate or worse non-proliferative DR or proliferative DR based on the International Clinical Diabetic Retinopathy Disease Severity Scale (ICDRDSS). Any DR included any type of DR including both non-referable and referable DR. CRVO was categorized separately from BRVO/hemi-CRVO. Other miscellaneous diseases such as retinal artery occlusion, retinal detachment, central serous chorioretinopathy, choroidal nevus, hemangioma, and myopic maculopathy, were conglomerated into “other” retinal/choroidal diseases/findings and considered abnormalities. Annotation guidelines are thoroughly described in Supplementary Table 4. Detailed information regarding the annotation processes and the analyses were elaborated in the previous publications^16,29,30^.

### Image preprocessing

The ocular fundus images were resized to 1024 × 1024 after cropping the black background to place the fovea in the center. Random augmentations were performed using rotation, flip, affine transform, image perturbation (color, contrast, brightness, sharpness, RGB shift, Gamma), noise (blur, ISO noise, Gaussian noise, JPEG compression, sunflare), resize, elastic transform, and down-sampling (1024 × 1024 image to 512 × 512 back to 1024 × 1024) to prevent the network from overfitting. Various Python packages (PIL, skimage, albumentation) were used to implement the augmentation methods. Validation and test images were not augmented.

### Target labels for training and standard reference annotations

For each fundus image, the target distribution used to compute the binary cross entropy loss when training the DL models were set using Naïve Bayes on annotations provided by the 3 readers. Because readers annotated the data independently without viewing others’ labels, the incidence of each finding and disease, as well as the sensitivities and specificities of each reader’s finding identification and diagnostic conclusions were treated as independent random variables. Empirical estimates were then used to derive the posterior probability of the reference label used for training. Specifically, the conditional probability $\Pr {(}Y_{k} = 1 {|}\hat{y}_{1,k} , \hat{y}_{2,k} , \hat{y}_{3,k} )$ that a fundus image indexed by $k$ contains a finding or disease $\left( {y_{k} = 1} \right)$ given 3 readers’ annotations $\left( {\hat{y}_{1,k} ,\hat{y}_{2,k} ,\hat{y}_{3,k} } \right)$, where each annotation describes the abnormality $y_{k} \in \left\{ {0,1} \right\}$, is given by

$$\frac{{\Pr \left( {\hat{Y}_{1,k} = \hat{y}_{1,k} {|}Y_{k} = 1} \right)\Pr \left( {\hat{Y}_{2,k} = \hat{y}_{2,k} {|}Y_{k} = 1} \right)\Pr \left( {\hat{Y}_{3,k} = \hat{y}_{3,k} {|}Y_{k} = 1} \right)\Pr \left( {Y_{k} = 1} \right)}}{{\mathop \sum \nolimits_{{y_{k} \in \left\{ {0,1} \right\}}} \Pr \left( {\hat{Y}_{1,k} = \hat{y}_{1,k} {|}Y_{k} = y_{k} } \right)\Pr \left( {\hat{Y}_{2,k} = \hat{y}_{2,k} {|}Y_{k} = y_{k} } \right)\Pr \left( {\hat{Y}_{3,k} = \hat{y}_{3,k} {|}Y_{k} = y_{k} } \right)\Pr \left( {Y_{k} = y_{k} } \right)}}$$

and can be computed using Naïve Bayes: $\Pr \left( { \cap_{a} \left\{ {\hat{Y}_{a,k} = \hat{y}_{a,k} } \right\}{|}Y_{k} = y_{k} } \right) = \mathop \prod \limits_{a} {\text{Pr}}(\hat{Y}_{a,k} = \hat{y}_{a,k} |Y_{k} = y_{k} ) \forall y$. The prior $\left( {\Pr \left( {Y_{k} = 0} \right),\Pr \left( {Y_{k} = 1} \right)} \right)$ along with the sensitivity and specificity of each annotator were estimated using the expectation–maximization algorithm^31^.

When readers disagreed on the disease severity, the annotations were assigned such that images with higher finding severity were also labeled positive for a less severe finding. In particular, the readers’ assessments for an image diagnosed with DR, referable DR, and no signs of DR, used to compute the target label for DR would be $\hat{y}_{DR} : = \left( {\hat{y}_{1,DR} ,\hat{y}_{2,DR} ,\hat{y}_{3,DR} } \right) = \left( {1,1,0} \right)$ whereas the annotations for computing the reference standard label for referable DR would be $\hat{y}_{DR} = \left( {0,1,0} \right)$. Images diagnosed with RVO and AMD were also processed in the same manner: if the annotations were BRVO/hemi-CRVO, CRVO, and no signs of RVO (similarly dry AMD, wet AMD, no AMD), the annotations for BRVO and CRVO would be $\hat{y}_{BRVO} = \left( {1,1,0} \right)$ and $\hat{y}_{CRVO} = \left( {0,1,0} \right)$ ($\hat{y}_{DryAMD} = \left( {1,1,0} \right), \hat{y}_{WetAMD} = \left( {0,1,0} \right)$).

Reference standard in the validation/test sets were chosen in a more conservative manner by assigning positive (abnormal) when the majority consensus was positive and negative when all readers unanimously submitted negative. Any image with only 1 positive and 2 negatives was excluded from the validation/test sets.

### Architectural design and training protocol

Fifteen finding identification networks $\left( {g_{1} ,w_{1} ,b_{1} } \right), \ldots ,\left( {g_{15} ,w_{15} ,b_{15} } \right)$, where $g_{f} :{\mathcal{X}} \to {\mathcal{Z}}$ maps a fundus image $x \in {\mathcal{X}} \subset {\text{R}}^{{\Omega }}$ with spatial resolution ${\Omega }$ to a latent space $z \in {\mathcal{Z}} \subset {\text{R}}^{{{\text{C}} \times {\Omega }^{\prime } }}$ and $\left( {w_{f} ,b_{f} } \right):\overline{z}_{f} \mapsto w_{f} \overline{z}_{f} + b_{f} \in {\text{R}}$ is the final fully-connected layer operating on global average pooled features $\overline{z} \in {\text{R}}^{C}$ before the sigmoid activation function $\sigma$ for prediction, were trained using the binary cross entropy loss as a multi-label classification task to separately identify each finding. The finding networks were composed of a universal encoder shared among the networks and individual branches for each finding.

Eight diagnosis networks $\left( {v_{1} ,c_{1} } \right), \ldots , \left( {v_{8} ,c_{8} } \right)$, each composed of a fully connected layer $\left( {v_{d} ,c_{d} } \right):\left( {\overline{z}_{1} , \ldots ,\overline{z}_{15} } \right) \mapsto \mathop \sum \limits_{f} v_{d,f}^{T} \overline{z}_{f} + c_{d} \in {\text{R}}$ followed by a sigmoid activation function $\sigma$ for the diagnostic prediction $\hat{y}_{d} = \sigma \left( {\mathop \sum \limits_{f} v_{d,f}^{T} \overline{z}_{f} + c_{d} } \right)$ on disease $d$, were then trained to diagnose fundus images from concatenated global average pooled features $\left( {\overline{z}_{1} , \ldots ,\overline{z}_{15} } \right)$ extracted at the penultimate layer of the finding networks.

In the implementation of the universal encoder and each finding branch, EfficientNet-B7^32^ was first modified to have 15 fully connected layers to classify all findings, and trained until the AUROC corresponding to different findings on the validation dataset began fluctuating as shown in Supplementary Fig. 3. In response to the observation that learning all findings’ features simultaneously results in conflicting gradients, separate branches were used for each finding inspired by previous works on multi-task learning^33–35^ while sharing low-level features to preserve information common in all findings. As visualized in the t-SNE plots^36^ at different layers (Supplementary Fig. 4), features are not distinguished clearly at shallow layers but form clusters at deeper layers. This is further quantified in Supplementary Fig. 5 by computing the mean cosine distance between pairs of findings $\left( {f_{i} , f_{j} } \right) \forall i \ne j$ computed as

$$d_{cos} \left( {f_{i} ,f_{j} } \right) = 1 - \frac{1}{{\left| {f_{i} } \right|\left| {f_{j} } \right|}}\mathop \sum \limits_{{I, J \in f_{i} \times f_{j} }} \frac{{{\Phi }\left( I \right)^{T} {\Phi }\left( J \right)}}{{\left\| {{\Phi }\left( I \right)} \right\|\left\| {{\Phi }\left( J \right)} \right\|}}$$

where ${\Phi }\left( I \right)$ denotes the global-average-pooled features extracted from image $I$ at the layer of interest and $\left| {f_{k} } \right|$ is the number of images with finding $k$. We found that the average cosine distance between finding pairs started to increase beyond ‘Block5a’ as in Supplementary Figs. 4 and 5, signifying that feature maps at ‘Block4a’ convey universal features informative of all findings whereas deeper layers learn discriminative features specific to each finding. We thus branched out after ‘Block4a’. The encoder was then frozen to fine-tune finding branches until the validation AUROC saturated. Experimentally, the significant performance was not observed between architectures of different size that that we chose to append top layers of B0, the smallest network in the parameter size for each finding branch (Supplementary Table 5). Experimentally, freezing the encoder has minor effect in the test and validation AUROC on the in-house dataset (Supplementary Fig. 6).

Target labels were assigned using the aforementioned Naïve Bayes and the model was trained using the sum of BCE and guidance loss^29^. Training samples were randomly sampled non-uniformly in batches of size 6 such that the expected number of positive and negative samples in a batch are equal. The B7-B0 network was trained using Nesterov-SGD, with initial learning rate set to 0.001 until the 9th epoch reduced by a factor of 10 at epoch 10, until the validation AUROC decreased. Both linear projection matrices were trained for a maximum of 10 epochs with batch size 64 using the same Nesterov-SGD with L2-regularization coefficient 0.0005. Other training details such as augmentation and sampling ratio were identical to that used for training the B7-B0 network. The learning curves are illustrated in Supplementary Fig. 7. In the experiment on predicting whether a fundus is normal, this final architecture did not show any degradation in AUROC compared to end-to-end models and other models with addition parameters (Supplementary Table 3).

### Quantifying clinical relations between finding-disease pairs

To understand how our DL-based CAD system infers its diagnostic predictions, CAR was defined as a measure of how much a specific finding contributed to diagnosing a certain disease by comparing its prediction with what its prediction would have been in a hypothetical situation in which the finding under consideration is present or absent. Before defining CAR, we first define instance-dependent counterfactual attribution which can be computed for all finding-disease pairs $\left( {f,d} \right)$ in a fundus image $x$. First notice how the finding features $\overline{z}_{f}$ can be decomposed as $\overline{z}_{f} = \overline{z}_{{f,||w_{f} }} \frac{{w_{f} }}{{\left\| {w_{f} } \right\|}} + \overline{z}_{{f, \bot w_{f} }}$ with a component $\overline{z}_{{f,||w_{f} }}$ parallel to $w_{f}$ and its orthogonal counterpart $\overline{z}_{{f, \bot w_{f} }}$:

$$\overline{z}_{f} = w_{f}^{T} \overline{z}_{f} \frac{{w_{f} }}{{\left\| {w_{f} } \right\|}} + \left[ {\overline{z}_{f} - w_{f}^{T} \overline{z}_{f} \frac{{w_{f} }}{{\left\| {w_{f} } \right\|}}} \right] = :\left( {\sigma^{ - 1} \left( {\hat{y}_{f} } \right) - b_{f} } \right)\frac{{w_{f} }}{{\left\| {w_{f} } \right\|}} + \overline{z}_{{f, \bot w_{f} }} .$$

The odds ${\mathcal{O}}\left( {d;x} \right)$ of disease $d$ given a fundus image $x$ is defined as the ratio between the model’s prediction for disease $d$ being present and absent:

$${\mathcal{O}}\left( {d;x} \right) \triangleq \frac{{\hat{y}_{d} }}{{1 - \hat{y}_{d} }} = {\text{exp}}\left( {\mathop \sum \limits_{f} v_{d,f}^{T} \overline{z}_{f} + c_{d} } \right).$$

Let the latent vector $\overline{z}_{\backslash f} = \left( {\sigma^{ - 1} \left( \epsilon \right) - b_{f} } \right)\frac{{w_{f} }}{{\left\| {w_{f} } \right\|}} + \overline{z}_{{f, \bot w_{f} }}$ be the hypothetical feature map had the feature corresponding to finding $f$ not been present in the image. The instance-dependent counterfactual attribution of finding $f$ in diagnosing disease $d$ from a fundus image $x$ is the odds after removing the diagnostic prediction’s dependency on finding $f$, hence its name counterfactual:

$${\mathcal{C}}\left( {f,d;x} \right) = \exp \left( {\mathop \sum \limits_{{f^{\prime} \ne f}} v_{{d,f^{\prime}}}^{T} \overline{z}_{{f^{\prime}}} + v_{d,f}^{T} \overline{z}_{\backslash f} + c_{d} } \right) .$$

For a finding-disease pair $\left( {f,d} \right)$ and finding prediction $\hat{y}_{f}$ on a fundus image $x$, the *instance-dependent* counterfactual attribution ratio (I-CAR) $R_{I - CAR} \left( {f,d;x} \right)$ is the ratio between the odds and the counterfactual attribution:

$${\mathcal{R}}_{I - CAR} \left( {f,d;x} \right): = \frac{{{\mathcal{O}}\left( {d;x} \right)}}{{{\mathcal{C}}\left( {f,d;x} \right)}} = \exp \left( {\left( {\sigma^{ - 1} \left( {\hat{y}_{f} } \right) - \sigma^{ - 1} \left( \epsilon \right)} \right)v_{d,f}^{T} \frac{{w_{f} }}{{\left\| {w_{f} } \right\|}}} \right),$$

where $\sigma^{ - 1}$ is the inverse sigmoid function and $\epsilon \in \left( {0, 1/100} \right)$ is a small positive number. If a user wishes to modify the attribution due to some finding prediction, the diagnostic prediction of diseases is modified accordingly by changing $\hat{y}_{f}$ in $\overline{z}_{f}$. This is useful when a user wants to reject model’s finding prediction in case of false positives and false negatives.

The two quantities described above establish the key intuition behind our main notion of CAR used to understand the decision-making process behind the DL-based CAD system. Replacing the prediction $\hat{y}_{f}$ in I-CAR with a high confidence of $1 - \epsilon$ is the finding-disease CAR, comparing two hypothetical situations in which a finding is present surely and absent with high confidence:

$${\mathcal{R}}_{CAR} \left( {f,d} \right) = \exp \left( {\left( {\sigma^{ - 1} \left( {1 - \epsilon } \right) - \sigma^{ - 1} \left( \epsilon \right)} \right)v_{d,f}^{T} \frac{{w_{f} }}{{\left\| {w_{f} } \right\|}}} \right).$$

The confidence level $\epsilon$ was chosen to be the 5-percentile ordered statistic of prediction values on benign cases in the in-house validation dataset.

Given a finding-disease pair, an attribution activation map that quantifies the influence of each finding $f$ in diagnosing disease $d$ can be visualized by modifying the class activation map^13^ as

$$A\left( {f,d;x} \right) = v_{d,f}^{T} \frac{{w_{f} }}{{\left\| {w_{f} } \right\|}}w_{f}^{T} g_{f} \left( x \right).$$

### Computation of odds ratios for human experts

Odds ratio of human experts were computed as following. Let $S$ and $I$ denote the set of annotator and image indices. Every image $x_{i}$ indexed by $i$ is associated with a finding $f_{i}^{s}$ and disease $d_{i}^{s}$ label indicating its presence of finding/diagnosis assigned by reader $s \in S$. All annotations were accumulated into a single $2 \times 2$ matrix $N$ as

$$N = \left( {\begin{array}{*{20}c} {\mathop \sum \limits_{j \in S} \mathop \sum \limits_{i \in I} 1\left\{ {f_{i}^{s} = 1{ } \wedge { }d_{i}^{s} = 1} \right\}} & {\mathop \sum \limits_{j \in S} \mathop \sum \limits_{i \in I} 1\left\{ {f_{i}^{s} = 1{ } \wedge { }d_{i}^{s} = 0} \right\}} \\ {\mathop \sum \limits_{j \in S} \mathop \sum \limits_{i \in I} 1\left\{ {f_{i}^{s} = 0 \wedge { }d_{i}^{s} = 1} \right\}} & {\mathop \sum \limits_{j \in S} \mathop \sum \limits_{i \in I} 1\left\{ {f_{i}^{s} = 0{ } \wedge { }d_{i}^{s} = 0} \right\}} \\ \end{array} } \right),$$

where $\wedge$ is the Boolean And operation and $1\left\{ \cdot \right\}$ is the indicator function. The odds ratio was then computed as

$$OR = \frac{{N_{0,0} N_{1,1} }}{{N_{0,1} N_{1,0} }}.$$

### External datasets

The proposed models were tested on 9 external datasets with their summary statistics described in Supplementary Table 6. Two datasets, MESSIDOR and STARE, contain both findings and disease annotations, whereas 2—e-ophtha and IDRiD-segmentation—contain only finding annotations and 5 contain disease annotations: Kaggle-APTOS (2019), IDRiD-classification, REFUGE (training), REFUGE (val, test), and ADAM.

MESSIDOR consists of 1200 macula-centered images taken by TOPCON TRC NW6 digital fundus camera [TOPCON, Tokyo, Japan] with a 45-degree field of view. The dataset provides DR grades in a scale of 4, from 0 to 3, which does not align with the 5-scale grading in ICDRDSS. Three retinal specialists (SJP, JYS, HDK) who participated in annotating the in-house data independently assessed the images in the dataset regarding 15 findings and 8 diseases and adjusted the annotations to be compatible with the ICDRDSS grading. Images considered ungradable by any of the 3 specialists were excluded from our study.

All the other 8 external datasets are public datasets available online. Assessments provided with the datasets were used as-is when the decisions were binary (present/absent or positive/negative). Annotations in ICDRDSS scales in Kaggle-APTOS and IDRiD—classification datasets were converted to binary annotations for DR for grades $\ge 1$ and referable DR for grades $\ge 2$. Binary labels in the ADAM dataset indicate the presence of AMD without subcategorizing as dry or wet AMD, and the two subcategories were grouped into a single AMD class with annotations assigned positive/present if either dry or wet AMD was present and absent otherwise. The higher of predictions on dry and wet AMD were used to evaluate the model. The laterality of an image was derived by the center of the optic disc using a segmentation network for optic disc^37^, e.g. right eye if the disc center is on the right side.

### Comparison with readers performance

To compare with human readers’ performance, 150 fundus images corresponding to 150 distinct subjects were sampled at the health screening center and ophthalmology outpatient clinic at SNUBH from July 1st, 2016 to June 30th, 2018. The images were captured with various fundus cameras including VX-10α, nonmyd 7, nonmyd WX [Kowa Optimed, Tokyo, Japan] with varying resolutions of (2144, 1424), (2992, 2000), (2464, 1632), (4288, 2848), and the data were annotated in disease names. Average age was 59.4 with standard deviation of 11.9 and there were 74 females (49.7%). The sampled data included cases of 25 DR (14 referable DR), 27 AMD (17 dry AMD, 10 wet AMD), 20 RVO (10 CRVO, 10 BRVO/hemi-CRVO), 13 glaucoma suspect, and 18 epiretinal membrane. We measured the performance of 4 physicians, and compared the performance with that of the DL algorithm. This dataset is denoted as ‘Reader Study’ and the operating point of each reader is shown in Fig. 2.

## Supplementary Information

Supplementary Information.

## Data Availability

Although public datasets are available at their respective repositories, SNUBH dataset is only available upon reasonable request to corresponding authors with the permission of the institution due to patient information protection law in Republic of Korea.

## Abbreviations

AMD: Age-related macular degeneration
AUROC: Area under the receiver operating characteristic curve
BRVO: Branch retinal vein occlusion
CAR: Counterfactual attribution ratio
CRVO: Central retinal vein occlusion
CWP: Cotton wool patch
DL: Deep learning
DNN: Deep neural network
DR: Diabetic retinopathy
ICDRDSS: International Clinical Diabetic Retinopathy Disease Severity Scale
IDRiD: Indian Diabetic Retinopathy image Dataset
REFUGE: REtinal FUndus Glaucoma challengE
RNFL: Retinal nerve fiber layer
RVO: Retinal vein occlusion
SNUBH: Seoul National University Bundang Hospital
t-SNE: t-distributed stochastic neighbor embedding

## Code availability

We used the scikit-learn (https://scikit-learn.org) and statsmodels (https://www.statsmodels.org) packages to conduct the statistical tests in computing the AUROCs using the exact Clopper-Pearson method and confidence intervals. The machine-learning models were developed by using standard model libraries and scripts in TensorFlow and Keras. Custom code was specific to our computing infrastructure and mainly used for data input/output and parallelization across computing nodes. Proprietary codes for training and the deployment of the CAD system are not publicly available but some boilerplate code may be available for research purposes from the corresponding author upon reasonable request.

## References

[1] Early Treatment Diabetic Retinopathy Study Research Group (1991). Grading diabetic retinopathy from stereoscopic color fundus photographs–an extension of the modified Airlie house classification ETDRS report number 10. Ophthalmology.

[2] Detry-Morel M (2004). Screening for glaucoma in a general population with the non-mydriatic fundus camera and the frequency doubling perimeter. Eur. J. Ophthalmol..

[3] Chew EY (2012). The age-related eye disease study 2 (AREDS2): Study design and baseline characteristics (AREDS2 report number 1). Ophthalmology.

[4] The Eye Disease Case-control Study Group (1993). Risk factors for branch retinal vein occlusion. Am. J. Ophthalmol..

[5] LeCun Y, Bengio Y, Hinton G (2015). Deep learning. Nature.

[6] Gulshan V (2016). Development and validation of a deep learning algorithm for detection of diabetic retinopathy in retinal fundus photographs. JAMA.

[7] Ting DSW (2017). Development and validation of a deep learning system for diabetic retinopathy and related eye diseases using retinal images from multiethnic populations with diabetes. JAMA.

[8] Li Z (2018). Efficacy of a deep learning system for detecting glaucomatous optic neuropathy based on color fundus photographs. Ophthalmology.

[9] Asaoka R, Murata H, Iwase A, Araie M (2016). Detecting preperimetric glaucoma with standard automated perimetry using a deep learning classifier. Ophthalmology.

[10] Burlina PM (2017). Automated grading of age-related macular degeneration from color fundus images using deep convolutional neural networks. JAMA Ophthalmol..

[11] Peng Y (2019). DeepSeeNet: A deep learning model for automated classification of patient-based age-related macular degeneration severity from color fundus photographs. Ophthalmology.

[12] Son J (2019). Development and validation of deep learning models for screening multiple abnormal findings in retinal fundus images. Ophthalmology.

[13] Zhou, B., Khosla, A., Lapedriza, A., Oliva, A. & Torralba, A. in *Proceedings of the IEEE Conference on Computer Vision and Pattern Recognition.* pp. 2921–2929.

[14] Selvaraju, R. R. *et al.* in *Proceedings of the IEEE International Conference on Computer Vision.* pp. 618–626.

[15] Sundararajan, M., Taly, A. & Yan, Q. in *Proceedings of the 34th International Conference on Machine *Learning, Vol. 70. 3319–3328 (JMLR. org).

[16] Park, S. J. *et al.* A novel fundus image reading tool for efficient generation of a multi-dimensional categorical image database for machine learning algorithm training. *J Korean Med Sci* **33** (2018). 10.3346/jkms.2018.33.e239 PMC6193885 30344460

[17] Decencière E (2014). Feedback on a publicly distributed image database: The Messidor database. Image Anal. Stereol..

[18] Decenciere E (2013). TeleOphta: Machine learning and image processing methods for teleophthalmology. Irbm.

[19] Prasanna Porwal, S. P. R. K., Manesh Kokare, Girish Deshmukh, Vivek Sahasrabuddhe and Fabrice Meriaudeau. (IEEE Dataport, 2018).

[20] Adam, H. *STARE database*, http://www.ces.clemson.edu/~ahoover/stare (2004).

[21] Orlando JI (2019). REFUGE challenge: A unified framework for evaluating automated methods for glaucoma assessment from fundus photographs. Med. Image Anal..

[22] Fu, H., Li, F., Orlando, J. I., Bogunović, H., Sun, X., Liao, J., Xu, Y., Zhang, S., Zhang, X. ADAM: Automatic Detection challenge on Age-related Macular degeneration (IEEE DataPort, 2020).

[23] Montavon G, Lapuschkin S, Binder A, Samek W, Müller K-R (2017). Explaining nonlinear classification decisions with deep Taylor decomposition. Patt. Recogn..

[24] Lundberg, S. & Lee, S.-I. A unified approach to interpreting model predictions. arXiv preprint http://arxiv.org/abs/1705.07874 (2017).

[25] Singh A, Sengupta S, Lakshminarayanan V (2020). Explainable deep learning models in medical image analysis. J. Imag..

[26] Qayyum A, Anwar SM, Awais M, Majid M (2017). Medical image retrieval using deep convolutional neural network. Neurocomputing.

[27] Lee H (2019). An explainable deep-learning algorithm for the detection of acute intracranial haemorrhage from small datasets. Nat. Biomed. Eng..

[28] Kim, B. *et al.* in *International Conference on Machine Learning.* 2668–2677 (PMLR).

[29] Son J, Bae W, Kim S, Park SJ, Jung K-H (2018). Computational Pathology and Ophthalmic Medical Image Analysis.

[30] Son, J., Kim, S., Park, S. J. & Jung, K-H. in *Intravascular Imaging and Computer Assisted Stenting and Large-Scale Annotation of Biomedical Data and Expert Label Synthesis: 7th Joint International Workshop, CVII-STENT 2018 and Third International Workshop, LABELS 2018, Held in Conjunction with MICCAI 2018, Granada, Spain, September 16, 2018, Proceedings 3.* 95–104 (Springer).

[31] Collins, M. The naive bayes model, maximum-likelihood estimation, and the em algorithm. *Lecture Notes* (2012).

[32] Tan, M. & Le, Q. V. EfficientNet: Rethinking Model Scaling for Convolutional Neural Networks. arXiv preprint http://arxiv.org/abs/1905.11946 (2019).

[33] Kendall, A., Gal, Y. & Cipolla, R. in *Proceedings of the IEEE Conference on Computer Vision and Pattern Recognition.* pp. 7482–7491.

[34] Teichmann, M., Weber, M., Zoellner, M., Cipolla, R. & Urtasun, R. in *2018 IEEE Intelligent Vehicles Symposium (IV).* pp. 1013–1020 (IEEE).

[35] Liao, Y., Kodagoda, S., Wang, Y., Shi, L. & Liu, Y. in *2016 IEEE international conference on robotics and automation (ICRA).* pp. 2318–2325 (IEEE).

[36] Maaten LVD, Hinton G (2008). Visualizing data using t-SNE. J. Mach. Learn. Res..

[37] Son J, Park SJ, Jung KH (2019). Towards accurate segmentation of retinal vessels and the optic disc in fundoscopic images with generative adversarial networks. J. Digit. Imaging.

