# Supplementary material for: An interpretable and interactive deep learning algorithm for a clinically applicable retinal fundus diagnosis system by modelling finding-disease relationship
Source: Sci Rep. 2023 Apr 12;13:5934. doi: 10.1038/s41598-023-32518-3 (PMC10097752; doi:10.1038/s41598-023-32518-3)
Supplement: Supplementary file 1 — Supplementary Information. [file 41598_2023_32518_MOESM1_ESM.docx]

**Supplementary Materials**

Supplementary Table 1, 2, 3, 4, 5, 6

Supplementary Figure 1, 2, 3, 4, 5, 6, 7

**Supplementary Table 1.** In-house data specifications. SNUBH dataset includes images of 18 different resolutions ranging from (1024, 1536) to (2848, 4288) The number of positive cases in the in-house training set is shown for more than 2 and more than 1 positive assessments out of 3 assessments.

|  | SNUBH dataset | | |  |
| --- | --- | --- | --- | --- |
|  |  | | |  |
| Total No. of fundus images | 103262 | | |  |
| Total No. of gradable images | 95,350 (92.3%) | | |  |
| Total No. of right eyes | 47586 | | |  |
| Total No. of left eyes | 47764 | | |  |
| No. of patients | 47026 | | |  |
| Age, mean (std) | 51.5±25.4 | | |  |
| Female, No (%) | 22,808 (48.5%) | | |  |
| Loc. of hospital | Korea | | |  |
| Camera | CF60Uvi, CR6-45NM, VX-10, VX-10α, nonmyd 7, GENESIS-D, etc | | |  |
| Image resolution (width, height) | 18 resolutions ranging from (1536, 1024) to (4288, 2848) | | |  |
| Number of Images (%) | Training set ≥2 pos [≥1 pos] | Tuning set | Test set |  |
|  |  |  |  |  |
| Normality |  |  |  |  |
| Abnormal Fundus | 24974 [36677] / 81036 (30.8 [45.3] %) | 1442 / 4807 (30.0 %) | 2911 / 9507 (30.6 %) |  |
| Finding |  |  |  |  |
| Hemorrhage | 3620 [5701] / 81036 (4.5 [7.0] %) | 208 / 4693 (4.4 %) | 453 / 9268 (4.9 %) |  |
| Hard Exudate | 1857 [3198] / 81036 (2.3 [3.9] %) | 85 / 4733 (1.8 %) | 243 / 9381 (2.6 %) |  |
| Cotton Wool Patch | 582 [1360] / 81036 (0.7 [1.7] %) | 41 / 4754 (0.9 %) | 92 / 9417 (1.0 %) |  |
| Drusen | 7373 [15000] / 81036 (9.1 [18.5] %) | 433 / 4360 (9.9 %) | 859 / 8609 (10.0 %) |  |
| Membrane | 3073 [5227] / 81036 (3.8 [6.5] %) | 169 / 4675 (3.6 %) | 316 / 9281 (3.4 %) |  |
| Macular Hole | 299 [772] / 81036 (0.4, [1.0] %) | 12 / 4779 (0.3 %) | 32 / 9462 (0.3 %) |  |
| Myelinated Nerve Fiber | 241 [401] / 81036 (0.3 [0.5] %) | 14 / 4801 (0.3 %) | 23 / 9490 (0.2 %) |  |
| Chorioretinal Atrophy | 2552 [5150] / 81036 (3.1 [6.4] %) | 138 / 4657 (3.0 %) | 317 / 9189 (3.4 %) |  |
| Vascular Abnormality | 586 [1732] / 81036 (0.7 [2.1] %) | 34 / 4729 (0.7 %) | 74 / 9379 (0.8 %) |  |
| RNFL defect | 1630 [4572] / 81036 (2.0 [5.6] %) | 83 / 4631 (1.8 %) | 189 / 9148 (2.1 %) |  |
| Glaucomatous Disc Change | 2972 [7982] / 81036 (3.7 [9.8] %) | 151 / 4506 (3.4 %) | 333 / 8896 (3.7 %) |  |
| Non-glaucomatous Disc Change | 918 [3702] / 81036 (1.1 [4.6] %) | 49 / 4647 (1.1 %) | 115 / 9156 (1.3 %) |  |
| Retinal Pigmentray Change | 2242 [8433] / 81036 (2.8 [10.4] %) | 151 / 4463 (3.4 %) | 260 / 8802 (3.0 %) |  |
| Fluid Accumulation | 352 [1443] / 81036 (0.4 [1.8] %) | 22 / 4757 (0.5 %) | 50 / 9383 (0.5 %) |  |
| Chorodial Lesion | 124 [1250] / 81036 (0.2 [1.5] %) | 6 / 4744 (0.1 %) | 15 / 9356 (0.2 %) |  |
| Diagnosis |  |  |  |  |
| Any DR | 2361 [3140] / 81036 (2.9 [3.9] %) | 131 / 4807 (2.7 %) | 274 / 9507 (2.9 %) |  |
| Referable DR | 1786 [2565] / 81036 (2.2 [3.2] %) | 87 / 4737 (1.8 %) | 210 / 9399 (2.2 %) |  |
| Dry AMD | 4448 [11447] / 81036 (5.5 [14.1] %) | 282 / 4393 (6.4 %) | 513 / 8705 (5.9 %) |  |
| Wet AMD | 589 [1534] / 81036 (0.7 [1.9] %) | 26 / 4751 (0.5 %) | 69 / 9386 (0.7 %) |  |
| BRVO / Hemi-CRVO | 382 [984] / 81036 (0.5 [1.2] %) | 19 / 4772 (0.4 %) | 55 / 9425 (0.6 %) |  |
| CRVO | 75 [165] / 81036 (0.1 [0.2] %) | 4 / 4799 (0.1 %) | 9 / 9491 (0.1 %) |  |
| Epiretinal Membrane | 2709 [4670] / 81036 (3.3 [5.8] %) | 157 / 4682 (3.4 %) | 278 / 9301 (3.0 %) |  |
| Glaucoma Suspect | 3735 [9469] / 81036 (4.6 [11.7] %) | 191 / 4483 (4.3 %) | 437 / 8848 (4.9 %) |  |

**Supplementary Table 2.**  Sensitivity and specificity of the proposed deep learning model on in-house and external test datasets. If no positive cases exist, the cells are filled with “-”. In case of ADAM dataset, which has no subcategory for AMD into dry AMD and wet AMD, we could not find a single operating point from in-house validation dataset as the prediction values were computed as higher prediction values between dry AMD and wet AMD.

|  | In-house test set | |  | External dataset (image) | |  | External datasets (image, annotation) | |
| --- | --- | --- | --- | --- | --- | --- | --- | --- |
|  | SNUBH test set | |  | MESSIDOR | |  |  |  |
|  |  |  |  |  |  |  |  |  |
|  | Sensitivity (%) | Specificity (%) |  | Sensitivity (%) | Specificity (%) |  | Sensitivity (%) | Specificity (%) |
| Normality |  |  |  |  |  |  |  |  |
| Abnormal Fundus | 88.0 (86.8-89.2) | 95.8 (95.2-96.3) |  | 71.9 (68.6-75.0) | 96.6 (94.4-98.1) |  | - | - |
| Finding |  |  |  |  |  |  |  |  |
| Hemorrhage | 98.0 (96.3-99.1) | 97.5 (97.1-97.8) |  | 83.8 (80.7-86.7) | 97.5 (95.9-98.7) |  | 85.1 (78.4-90.4) [e-ophtha] 100.0 (93.4-100.0) [IDRiD] | 98.7 (96.3-99.7) [e-ophtha],  70.8 (60.2-79.9) [IDRiD] |
| Hard Exudate | 98.4 (95.8-99.5) | 97.2 (96.8-97.5) |  | 92.3 (88.5-95.1) | 88.3 (86.0-90.3) |  | 95.7 (85.5-99.5) [e-ophtha] 100.0 (93.4-100.0) [IDRiD] | 65.7 (47.8-80.9) [e-ophtha],  96.6 (90.5-99.3) [IDRiD] |
| Cotton Wool Patch | 98.9 (94.1-100.0) | 99.1 (98.9-99.3) |  | 68.9 (61.9-75.3) | 94.9 (93.3-96.2) |  | 92.3 (74.9-99.1) [IDRiD] | 96.6 (91.5-99.1) [IDRiD] |
| Drusen | 93.8 (92.0-95.3) | 95.2 (94.7-95.7) |  | 42.5 (34.7-50.6) | 98.0 (96.9-98.7) |  | - | - |
| Membrane | 99.1 (97.3-99.8) | 96.4 (96.0-96.8) |  | 67.6 (49.5-82.6) | 93.3 (91.7-94.7) |  | - | - |
| Macular Hole | 96.9 (83.8-99.9) | 97.4 (97.0-97.7) |  | 100.0 (2.5-100.0) | 97.8 (96.8-98.6) |  | - | - |
| Myelinated Nerve Fiber | 100.0 (85.2-100.0) | 100.0 (99.9-100.0) |  | 100.0 (47.8-100.0) | 100.0 (99.7-100.0) |  | 100.0 (29.2-100.0) [STARE] | 98.7 (97.1-99.6) [STARE] |
| Chorioretinal Atrophy | 98.7 (96.8-99.7) | 96.5 (96.1-96.9) |  | 73.7 (60.3-84.5) | 97.7 (96.7-98.5) |  | - | - |
| Vascular Abnormality | 97.3 (90.6-99.7) | 96.8 (96.4-97.1) |  | 82.6 (61.2-95.0) | 91.0 (89.2-92.6) |  | - | - |
| RNFL defect | 97.9 (94.7-99.4) | 94.3 (93.8-94.8) |  | 69.6 (47.1-86.8) | 95.5 (94.1-96.6) |  | - | - |
| Glaucomatous Disc Change | 92.5 (89.1-95.1) | 93.4 (92.8-93.9) |  | 88.9 (51.8-99.7) | 96.2 (94.9-97.2) |  | - | - |
| Non-glaucomatous Disc Change | 93.0 (86.8-96.9) | 94.2 (93.7-94.7) |  | 50.0 (15.7-84.3) | 94.8 (93.4-96.0) |  | - | - |
| Retinal Pigmentray Change | 95.8 (92.6-97.9) | 88.3 (87.6-88.9) |  | 46.0 (31.8-60.7) | 97.5 (96.4-98.3) |  | - | - |
| Fluid Accumulation | 94.0 (83.5-98.7) | 93.8 (93.3-94.3) |  | 54.5 (23.4-83.3) | 96.9 (95.7-97.8) |  | - | - |
| Chorodial Lesion | 100.0 (78.2-100.0) | 95.3 (94.8-95.7) |  | 50.0 (27.2-72.8) | 93.6 (92.0-94.9) |  | - | - |
| Diagnosis |  |  |  |  |  |  |  |  |
| Any DR | 98.5 (96.3-99.6) | 97.2 (96.9-97.6) |  | 83.3 (80.1-86.3) | 97.5 (96.0-98.6) |  | 98.5 (97.8-99.0) [APTOS], 95.4 (92.6-97.3) [IDRiD] | 98.4 (97.7-98.9) [APTOS],  69.0 (61.5-75.9) [IDRiD] |
| Referable DR | 99.0 (96.6-99.9) | 98.1 (97.8-98.4) |  | 87.9 (84.3-90.9) | 96.1 (94.5-97.4) |  | 98.9 (98.3-99.4) [APTOS], 94.7 (91.7-96.9) [IDRiD] | 84.5 (82.9-86.0) [APTOS],  84.5 (78.6-89.3) [IDRiD] |
| Dry AMD | 95.8 (93.7-97.3) | 92.9 (92.3-93.4) |  | 66.0 (50.7-79.1) | 96.8 (95.6-97.7) |  | - | - |
| Wet AMD | 100.0 (94.8-100.0) | 95.9 (95.5-96.3) |  | - | - |  |  |  |
| BRVO / Hemi-CRVO | 88.1 (77.1-95.1) | 98.3 (98.0-98.6) |  | 100.0 (15.8-100.0) | 89.9 (88.0-91.6) |  | 100.0 (85.2-100.0) [STARE] | 77.3 (72.7-81.4) [STARE] |
| CRVO | 88.9 (51.8-99.7) | 99.9 (99.9-100.0) |  | - | - |  | 79.2 (57.8-92.9) [STARE] | 93.8 (90.9-96.1) [STARE] |
| Epiretinal Membrane | 99.3 (97.4-99.9) | 96.8 (96.4-97.2) |  | 81.2 (54.4-96.0) | 93.3 (91.7-94.6) |  | 100.0 (15.8-100.0) [STARE] | 83.5 (79.5-87.1) [STARE] |
| Glaucoma Suspect | 93.1 (90.3-95.3) | 91.8 (91.2-92.3) |  | 77.8 (52.4-93.6) | 94.9 (93.5-96.1) |  | 82.5 (67.2-92.7) [REFUGE - train],  85.0 (75.3-92.0) [REFUGE-val,test] | 96.7 (94.2-98.3) [REFUGE - train],  97.8 (96.4-98.7) [REFUGE-val,test] |

**Supplementary Table 3.** In-house validation and test AUROCs for different architectures classifying abnormalities in a fundus image. ‘Linear projection’ refers to our final model which maps concatenated finding features to the binary decision for normality. The suffix ‘+ B0’ refers that an additional B0 was appended to the concatenated finding features. The B7-B0 model was trained in an end-to-end manner with frozen and fine-tuned B7 blocks. All results are obtained within a 95% confidence interval. Training a linear projection matrix mapping the extracted features to 15 major findings suffices in determining abnormalities in a fundus image.

|  | Architecture | | | |  |
| --- | --- | --- | --- | --- | --- |
|  | Linear projection | Linear projection (frozen) + B0 | Linear projection  (fine-tuned) + B0 | B7 (frozen) - B0 | B7 (fine-tuned) - B0 |
| Val AUROC  (best) | 97.6 (96.5-98.4) | 97.5 (96.4-98.2) | 97.6 (96.5-98.4) | 97.6 (96.5-98.4) | 97.6 (96.6-98.4) |
| Test AUROC | 97.7 (96.7-98.5) | 97.7 (96.6-98.4) | 97.8 (96.7-98.5) | 97.9 (96.9-98.6) | 97.9 (96.9-98.6) |

**Supplementary Table 4.** Summary of annotation guidelines.

| Category | Definition |
| --- | --- |
| Findings |  |
| Hemorrhage | All types of hemorrhage including preretinal, retinal, vitreous, subretinal, disc, microaneurysm, and dot hemorrhage |
| Hard exudate | Hard exudate of all shapes and textures |
| Cotton wool patch | Cotton wool spots, or soft exudate, of various shapes |
| Drusen | All types of drusen including hard drusen, soft drusen, reticular pseudodrusen, and drusenoid deposit |
| Membrane | All types of retinal membrane including epiretinal membrane and tractional retinal detachment |
| Macular hole | Foveal detachment, partial-thickness holes, and full-thickness holes |
| Myelinated nerve fiber | Myelination in retinal nerve fiber layer of any size |
| Chorioretinal atrophy | All types of chorioretinal atrophy including Sveinsson chorioretinal atrophy and progressive bifocal chorioretinal atrophy |
| Vascular abnormality | All types of vascular abnormality which include retinal vein occlusion, retinal artery occlusion, ghost vessel, collaterals, neovascularization |
| Retinal nerve fiber layer defect | Any visible loss in retinal nerve fiber layer |
| Glaucomatous disc change | ISNT rule violation, Rim narrowing/notching, acquired optic nerve pit |
| Non-glaucomatous disc change | Optic neuropathy excluding glaucoma such as pale disc, papilledema |
| Retinal pigmentary change | Retinal Pigment Epithelium (RPE) hyperpigmentation, RPE depigmentation |
| Fluid accumulation | Subretinal fluid, intraretinal fluid, macular edema, pigment epithelial detachment |
| Chorodial lesion | Nevus, elevation |
| Diagnosis |  |
| Any Diabetic Retinopathy | Mild and worse DR in ICDRDSS |
| Referable DR | Moderate and worse DR in ICDRDSS |
| Dry Age-related Macular Degeneration | Early AMD and geographic atrophy |
| Wet AMD | Severe AMD with fluid or blood in the macular area |
| Central Retinal Vein Occlusion | Ischemic and non-ischmic CRVO |
| Branch RVO / Hemi-CRVO | Ischemic and non-ischmic BRVO and Hemi-CRVO  (partial occlusion at the anterior part of the central retinal artery trunk) |
| Epiretinal Membrane | Fovea-involving and fovea-sparing epiretinal membrane |
| Glaucoma Suspect | Fundus suspicious of glaucoma that needs further examination |

**Supplementary Table 5.** In-house validation performances (AUROC) for each finding and average train loss (BCE) for different top-layer architectures using an identical encoder. The average AUROC does not vary considerably among different architectures although the AUROC for individual findings favor different architectures. We chose to use B0 for the top layers of each branch because it has the lowest number of parameters without much performance degradation.

|  | Architecture for top layers | | | | | |
| --- | --- | --- | --- | --- | --- | --- |
| Findings | B0 | B1 | B2 | B3 | B4 | B7 |
| Hemorrhage | 98.0 | 98.2 | 98.3 | 99.1 | 98.1 | 93.9 |
| Hard exudate | 99.8 | 99.7 | 99.8 | 99.8 | 99.8 | 99.3 |
| Cotton wool patch | 99.5 | 99.7 | 99.4 | 99.0 | 98.3 | 98.4 |
| Drusen | 98.3 | 98.6 | 98.2 | 97.9 | 98.0 | 97.8 |
| Membrane | 97.5 | 98.4 | 97.9 | 98.5 | 98.1 | 97.9 |
| Macular hole | 96.6 | 98.6 | 97.3 | 99.4 | 94.4 | 99.4 |
| Myelinated nerve fiber | 99.9 | 100.0 | 100.0 | 100.0 | 99.9 | 97.0 |
| Chorioretinal atrophy | 99.1 | 99.8 | 99.5 | 99.6 | 99.1 | 99.4 |
| Vascular abnormality | 95.2 | 98.6 | 98.0 | 98.9 | 97.3 | 94.1 |
| Reitnal nerve fiber layer defect | 93.6 | 95.0 | 95.1 | 90.9 | 93.5 | 91.9 |
| Glaucomatous disc change | 96.0 | 96.2 | 96.6 | 94.2 | 96.1 | 95.8 |
| Non-glaucomatous disc change | 93.3 | 87.2 | 91.6 | 89.4 | 94.4 | 91.6 |
| Retinal pigmentray change | 92.8 | 94.1 | 93.6 | 92.8 | 91.4 | 91.2 |
| Fluid accumulation | 96.2 | 98.6 | 93.4 | 96.9 | 95.6 | 95.4 |
| Chorodial lesion | 89.7 | 87.7 | 91.4 | 93.0 | 89.7 | 96.7 |
| Average AUROC | 96.4 | 96.7 | 96.7 | 96.6 | 96.2 | 96.0 |
| Minimum AUROC | 89.7 | 87.2 | 91.4 | 89.4 | 89.7 | 91.2 |
| Average BCE loss | 0.473 | 0.243 | 0.299 | 0.483 | 0.497 | 0.351 |
| Trainable Parameters | 4,960,581 | 7,423,605 | 8,587,747 | 11,554,757 | 18,304,161 | 63,786,960 |

**Supplementary Table 6**. External data specifications.

|  | External image |  | External image, External annotation | | | | | | | |
| --- | --- | --- | --- | --- | --- | --- | --- | --- | --- | --- |
|  | MESSIDOR |  | e-ophtha | APTOS | IDRiD - segmentation | IDRiD - classification | REFUGE (training) | REFUGE (val, test) | ADAM | STARE |
|  |  |  |  |  |  |  |  |  |  |  |
| Total No. of fundus images | 1200 |  | 434 | 3662 | 143 | 516 | 400 | 800 | 400 | 397 |
| Total No. of gradable images | 1,189 (99.1%) |  | 434 (100.0%) | 3,662 (100.0%) | 143 (100.0%) | 516 (100.0%) | 400 (100.0%) | 800 (100.0%) | 400 (100.0%) | 397 (100.0%) |
| Total No. of right eyes | 601 |  | 219 | 1914 | 70 | 253 | ~200 | ~400 | ~200 | 198 |
| Total No. of left eyes | 588 |  | 215 | 1745 | 73 | 263 | ~200 | ~400 | ~200 | 199 |
| No. of patients | N/A |  | 203 | N/A | N/A | N/A | N/A | N/A | 400 | N/A |
| Age, mean (std) | N/A |  | N/A | N/A | N/A | N/A | N/A | 25.3±11.5 | N/A | N/A |
| Female, No (%) | N/A |  | N/A | N/A | N/A | N/A | N/A | N/A (~52%) | N/A | N/A |
| Loc. of hospital | France |  | France | India | India | India | China | China | China | U.S. |
| Camera | TOPCON TRC NW6 |  | N/A | N/A | Kowa VX-10α | Kowa VX-10α | Zeiss Visucam 500 | Canon CR-2 | Zeiss Visucam 500 | Film slide |
| Image resolution (width, height) | (1440,960), (2240,1488), (2304,1536) |  | (2544, 1696), (2048, 1360),  (1440, 960), (1504, 1000) | 17 resolutions rainging from (640, 480) to (4288, 2848) | (4288, 2848) | (4288, 2848) | (2124, 2056) | (1634, 1634) | (2124, 2056) | N/A |
| Normality |  |  |  |  |  |  |  |  |  |  |
| Abnormal Fundus | 775 / 1189 (65.2%) |  | - | - | - | - | - | - | - | - |
| Finding |  |  |  |  |  |  |  |  |  |  |
| Hemorrhage | 619 / 1189 (52.1%) |  | - | - | 53 / 143 (37.0%) | - | - | - | - | - |
| Microaneurysms | - |  | 148 / 381 (38.8%) | - | 54 / 143 (37.7%) | - | - | - | - | - |
| Hard Exudate | 284 / 1189 (23.9%) |  | 47 / 82 (57.3%) | - | 54 / 143 (37.7%) | - | - | - | - | - |
| Cotton Wool Patch | 196 / 1189 (16.5%) |  | - | - | 26 / 143 (18.1%) | - | - | - | - | - |
| Drusen | 160 / 1189 (13.5%) |  | - | - | - | - | - | - | - | - |
| Membrane | 34 / 1189 (2.9%) |  | - | - | - | - | - | - | - | - |
| Macular Hole | 1 / 1189 (0.1%) |  | - | - | - | - | - | - | - | - |
| Myelinated Nerve Fiber | 5 / 1189 (0.4%) |  | - | - | - | - | - | - | - | 3 / 397 (0.8%) |
| Chorioretinal Atrophy | 57 / 1189 (4.8%) |  | - | - | - | - | - | - | - | - |
| Vascular Abnormality | 23 / 1189 (1.9%) |  | - | - | - | - | - | - | - | - |
| RNFL defect | 23 / 1189 (1.9%) |  | - | - | - | - | - | - | - | - |
| Glaucomatous Disc Change | 9 / 1189 (0.8%) |  | - | - | - | - | - | - | - | - |
| Non-glaucomatous Disc Change | 8 / 1189 (0.7%) |  | - | - | - | - | - | - | - | - |
| Retinal Pigmentray Change | 50 / 1189 (4.2%) |  | - | - | - | - | - | - | - | - |
| Fluid Accumulation | 11 / 1189 (0.9%) |  | - | - | - | - | - | - | - | - |
| Chorodial Lesion | 20 / 1189 (1.7%) |  | - | - | - | - | - | - | - | - |
| Diagnosis |  |  |  |  |  |  |  |  |  |  |
| Any DR | 582 / 1189 (48.9%) |  | - | 1857 / 3662 (50.7%) | - | 348 / 516 (67.4%) | - | - | - | - |
| Referable DR | 412 / 1189 (34.7%) |  | - | 1487 / 3662 (40.6%) | - | 323 / 516 (62.6%) | - | - | - | - |
| Dry AMD | 47 / 1189 (4%) |  | - | - | - | - | - | - | 89 / 400 (22.25%) | - |
| Wet AMD | 0 / 1189 (0%) |  | - | - | - | - | - | - |  | - |
| BRVO / Hemi-CRVO | 2 / 1189 (0.2%) |  | - | - | - | - | - | - | - | 23 / 397 (5.8%) |
| CRVO | 0 / 1189 (0%) |  | - | - | - | - | - | - | - | 24 / 397 (6.0%) |
| Epiretinal Membrane | 16 / 1189 (1.3%) |  | - | - | - | - | - | - | - | 2 /397 (0.5%) |
| Glaucoma Suspect | 18 / 1189 (1.5%) |  | - | - | - | - | 40 / 400 (10.0%) | 80 / 800 (10.0%) | - | - |

**Supplementary Figure 1.** ROC curves for findings and abnormality. Gray boxes, which span [-0.03, 0.3]×[0.7, 1.03] in the XY plane, are amplified to present the profile of the curves in a practical range. Operating points are shown with circles. CWP: Cotton Wool Patch, RNFL: Retinal Nerve Fiber Layer.

**Supplementary Figure 2**. Application of CAR to chest radiographs. **A**: relationship between radiologic findings with pulmonary diseases computed by CAR. **B**: heatmaps of attributing radiographic findings. **C:** Receiver Operating Characterisitic curves for classification of pulmonary diseases with captions indicating disease name (training data → test data) [AUROC]. A DNN was first trained to classify 7,104 normal and 7,821 abnormal chest X-ray radiographs with radiographic findings of nodule, consolidation, interstitial opacity, pleural effusion, pneumothorax. Then, the feature maps for the radiographic findings were concatenated and appended logistic regression layers to identify the onset of pulmonary diseases: tuberculosis (Shenzen and Montgomery), edema (CheXpert), and pneumonia (Shenzen, Montogomery, and RSNA Pneumonia challenge).


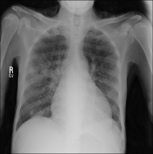

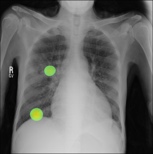


Pneumonia

(RSNA)


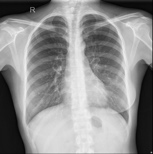

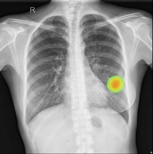


Tuberculosis

(Shenzhen)

Tuberculosis

(Montgomery)

Edema

(Chexpert)


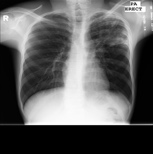

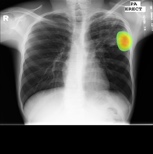

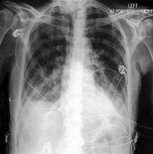

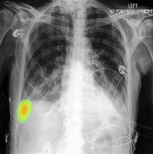


Original Image

Top 1 Finding

Top 2 Finding


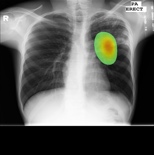


**A**

**B**

**C**

**Supplementary Figure 3.** Validation AUROC (left) and training loss (right) for 15 findings. The validation performances fluctuate in discordance instead of improving jointly.

**Supplementary Figure 4.** t-SNE plots of fundus images with global-average-pooled feature maps extracted at different layers. Images with multiple findings were excluded to emphasize the difference between feature maps of distinct findings. Layers progressively become deeper from ‘Block4a’ to ‘Block7a’. Features extracted at ‘Block7a’ cluster by findings, and several findings such as drusen, membrane, and chorioretinal atrophy begin to cluster at ‘Block5a’.

**Supplementary Figure 5.** Mean cosine distance at different convolution layers. The cosine distance tends to increase after ‘Block4a’ indicating that deeper layers learn more discriminative features specific to distinct findings.

**Supplementary Figure 6.** Performance of the B7-B0 network with different blocks frozen for RNFL Defect. Frozen block on the x axis indicates that all parameters were frozen from input to the given block. Models with the highest in-house validation AUROC were evaluated on the in-house test set. Models with weights up to blocks 3, 4, and 5 frozen performed better (test AUROC) when shallow layers were frozen. The performance decreased rapidly when layers up to block 6 and 7 were frozen due to a lack of trainable parameters.

**Supplementary Figure 7.** Training loss and validation AUROC for each finding, diagnosis, and abnormality by epoch. The network was trained until the validation AUROCs began oscillating for all findings. Only BCE loss is shown for the findings’ plots. First epoch is marked with index 0 on the x-axis.
